# *Henckelia siangensis* (Gesneriaceae): a remarkable new species from Northeast India

**DOI:** 10.3897/phytokeys.160.54459

**Published:** 2020-09-08

**Authors:** Momang Taram, Dipankar Borah, Ojar Taku, Hui Tag

**Affiliations:** 1 Department of Botany, Rajiv Gandhi University, Rono Hills, Doimukh, 791112, Arunachal Pradesh, India Rajiv Gandhi University Doimukh India; 2 Department of Botany, Goalpara College, Goalpara 783 101, Assam, India Goalpara College Goalpara India; 3 Geku, Upper Siang district, 791002, Arunachal Pradesh, India Unafiliated Geku India

**Keywords:** Arunachal Pradesh, flora of India, Himalaya, morphology, new taxon

## Abstract

*Henckelia siangensis*, a new species from Arunachal Pradesh of Northeast India, is described and illustrated here. The new species is remarkably different from all other allied species by its 5-winged calyx and elliptic-ovate calyx segments. It superficially resembles *H. calva* in glabrous stem and petioles, but differs from it in having persistent bracts, a campanulate 5-winged calyx and a style with glandular indumentum. A detailed morphological description, photographic illustration, and distribution of the new species are presented.

## Introduction

*Henckelia* Spreng. is a tropical genus of the family Gesneriaceae, comprising about 70 species (Kanthraj et al. 2020). The genus is distributed in the India, Bangladesh, Nepal, Bhutan, China, Myanmar, Sri Lanka, Thailand, Laos and Vietnam (Kanthraj et al. 2020). In India, the genus is so far represented by 35 species, 19 of them occurring in the Himalayas and Northeast India (Krishna and Lakshminarasimhan 2018; Borah et al. 2019; Kanthraj et al. 2020). The species of Northeast India along with others found in Sri Lanka were formerly attributed to Chirita sect. Chirita (Möller et al. 2017). They are all characterized by a caulescent habit, leaves in whorls of 2 or 3, orthocarpic capsules dehiscing along both the upper and lower sutures and unappendaged seeds (Möller et al. 2017). During our trips to the East Siang district of Arunachal Pradesh in April of 2019, a few interesting specimens of *Henckelia* were collected. They were critically studied, consulting the type specimens housed in several herbaria and scrutiny of relevant literature. The studies revealed that our specimen is remarkably different from all other species of *Henckelia* and led us to conclude that it represents a new species. The striking yellow flowers found in this species are fairly uncommon as only three other species of *Henckelia* with yellow flowers are known from NE India (*H. pathakii*, *H. calva* and *H. dimidiata*) and two others from China (*H. shuii* and *H. xinpingensis*). But as stated by Wood (1974), corolla color in *Henckelia* can vary considerably within a single species due to edaphic factors, hence much emphasis on the corolla color is not given here. A diagnostic key to the yellow flowered species of *Henckelia* in Northeast India is presented below for easy identification. Comparison of this peculiar new species was made with a superficially allied species *H. calva*, in the glabrous nature of the stem and petioles and lanceolate bracteoles. This new species is described and illustrated here.

## Methods

Flowering stems were collected from the field and photographed using a digital camera (Nikon COOLPIX B600, Nikon India Pvt. Ltd). GPS coordinates were recorded using Garmin GPS (Etrex 10 device, Asim Navigation India Pvt. Ltd). All collected specimens were processed using standard herbarium methods (Jain and Rao 1977) and voucher specimens were deposited in ASSAM and ARUN. Morphological observations and measurements of the new species were made on both freshly collected and dried specimens. The micro morphological characters were studied with a stereomicroscope (Leica S8APO, Leica Microsystems Inc., Germany) and were compared with those reported in the relevant literature (Clarke 1874; Clarke 1883; Hooker 1885; Chatterjee 1948; Wood 1974; Burtt et al. 1988; Weitzman et al. 1997; Wang et al. 1998; Weber et al. 2011; Middleton et al. 2013; Sinha and Datta 2016; Möller et al. 2017; Krishna and Lakshminarasimhan 2018; Borah et al. 2019; Cai et al. 2019; Sirimongkol et al. 2019; Yang et al. 2019; Bui et al. 2020; Janeesha and Nampy 2020; Kanthraj et al. 2020) and digital images of type specimens present at K, E and PE, as well as actual sheets housed at ASSAM, ARUN and CAL.

## Taxonomic treatment

### Henckelia siangensis

Taxon classificationPlantaeLamialesGesneriaceae

Taram, D.Borah & Tag sp. nov.

96252C2D-77FF-5536-AD18-A57FC51F39B2

urn:lsid:ipni.org:names:77211420-1

#### Type.

**Figure 1.**
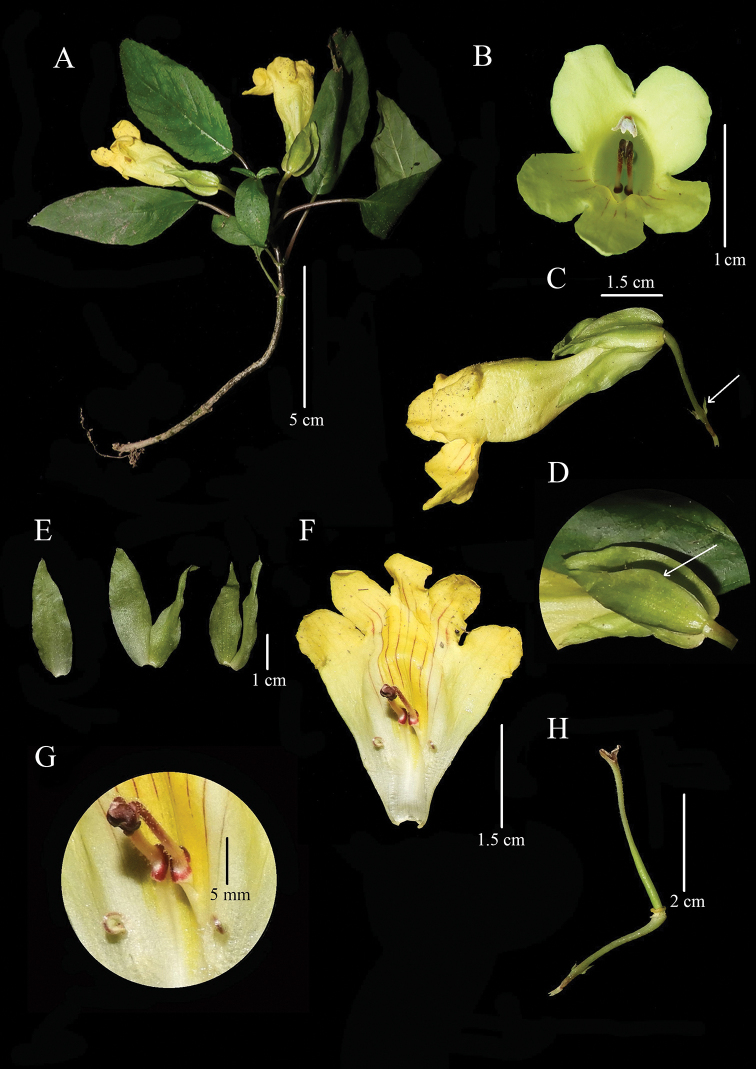
*Henckelia siangensis*. **A** flowering stem **B** flower front view **C** flower side view (arrow indicates the bracteoles) **D** winged calyx (arrow indicates the wings) **E** dissected calyx segments **F** dissected corolla **G** stamens and staminodes **H** pistil (Photographs by Momang Taram).

India. Arunachal Pradesh: East Siang District, Pasighat, 28°13'54"N, 95°13'19"E; 375 m asl., 26 April 2019, *Ojar Taku* and *Momang Taram* 05001 (holotype: ASSAM; isotype: ARUN). (Fig. 1)

#### Diagnosis.

The species is remarkably different from all other species of *Henckelia* by its 5-winged calyx and elliptic-ovate calyx segments. It is superficially similar to *H. calva* (C.B.Clarke) D.J.Middleton & Mich.Möller in glabrous stem and petiole as well as lanceolate bracts, but can be easily distinguished by ovate to lanceolate lamina with denticulate to serrulate margins (vs. elliptic to oblong- elliptic with entire to sub-entire and ciliate margin), peduncle 0.4–0.8 cm, glabrous (vs. 2–3 cm long, glabrescent), bracts persistent (vs. deciduous), calyx segments elliptic to ovate, raised at margins forming wings (vs. narrowly triangular to lanceolate), corolla glandular pubescent inside (vs. glabrous) and glandular hairy style (vs. puberulent).

Erect perennial herb, caulescent, 15–40 cm high, stems branched; mature stem terete, ca. 0.5 cm across, glabrous, young stem reddish brown, cylindrical. Leaves decussate; petiole terete, glabrous, slightly ridged at base, 1–6 × 0.1 cm, fleshy; lamina ovate to lanceolate, 7–10 × 2.5–4.5 cm, oblique–obtuse base, acute to shortly acuminate at apex, margin denticulate–serrulate, sparsely hirsute on both surfaces, hairs hyaline, dark green above, pale green below; secondary veins 6–10 pairs, sub-opposite, obscure above, raised beneath. Inflorescence axillary, 1 flowered cymes; peduncle 0.4–0.8 cm long, glabrous; pedicel glabrous 1.8–2.3 long, ca. 0.1cm thick; bracteoles 2, glabrous, green, opposite to sub opposite, ovate to lanceolate, 4–7 × 2–4 mm, apex acute, margin entire to sub entire. Calyx green, campanulate, 5 winged, raised at the fusion of the lobes, splitting with maturity, later turning 5 lobed up to below middle of the tube, glabrous, veins obscure, tube 0.6–0.7 cm long; calyx segments 2.0–2.3 × 0.6–1.1 cm, elliptic–ovate, margin entire, apex acute. Corolla 4.5–5 × 1.4–1.8 cm, bright yellow with three dark yellow and maroon stripes per lobe near throat, more prominent on lower lip, glabrescent outside, glandular pubescent inside (dense within tube), distinctly 2 lipped, lips divergent; upper lip 2-lobed, equal, broadly ovate, 0.4–0.6 cm × 0.5–0.7 cm, apex round, margin entire; lower lip 3-lobed, lobes sub-equal, broadly ovate, 0.7–1 × 0.5–0.6 cm, apex round, margin entire; tube 2.8–3.5 × 1.3–1.7 cm. Stamens 2, inserted 2–2.3 cm above corolla base, anthers and top of filaments maroon, glandular pubescent; anthers 0.2–0.3 × 0.1–0.2 cm, cohering face to face; filaments 0.8–1.5 × 0.1 cm, geniculate near base, knee dark pink-maroon. Staminodes 3, lateral staminodes 2, divergent (sometimes coiled), 0.4–0.7 cm long, hirsute, maroon, central staminode white, antherodes white-green, 0.2–0.3 cm long. Pistil 2.8–3.6 cm long; style glandular hairy, 1.5– 1.9 cm long; stigma chiritoid, lower lip 2 lobed, lobe apex acute to obtuse; disc yellow, undulate–annular, ca. 0.1 cm high. Ovary green, glabrous to glabrescent, 1.6–2 × 0.15 cm. Capsules not seen.

#### Phenology.

Flowering: April and fruiting: September.

#### Etymology.

The species is named after the type locality, the Siang valley.

#### Vernacular name

**(assigned here).***Libe lirak Appun* (in Adi language)

#### Distribution.

So far only known from East Siang district of Arunachal Pradesh, India.

#### Habitat and ecology.

It grows in moist shady places in rock crevices in association with *Henckelia mishmiensis* (Debb. ex Biswas) D.J.Middleton & Mich.Möller, *Aeschynanthus superbus* C.B.Clarke, *Begonia josephii* A.DC., *B. burkillii* Dunn., *Elatostema sessile* J.R.Forst. & G.Forst., *Pilea umbrosa* Blume, *Nephrolepis cordifolia* (L.) C.Presl., *Selaginella* sp. etc.

### Key to yellow species of *Henckelia* in Northeast India

**Table:** 

1	Bracts cupular, concealing pedicel of flower buds	***H. pathakii***
–	Bracts free, not concealing pedicel of flower buds	**2**
2	Bracts denticulate, calyx sub-equal and acuminate	***H. dimidiata***
–	Bracts entire, calyx equal and acute	**3**
3	Bracts deciduous, calyx tubular, not-winged, triangular to lanceolate, style pubescent	***H. calva***
–	Bracts persistent, calyx campanulate, winged, segments elliptic – ovate, style glandular hairy	***H. siangensis***

## Supplementary Material

XML Treatment for Henckelia siangensis
